# Feasibility study of shutter scan acquisition for region of interest (ROI) digital tomosynthesis

**DOI:** 10.1002/acm2.12294

**Published:** 2018-03-01

**Authors:** Dohyeon Kim, Byungdu Jo, Donghoon Lee, Haenghwa Lee, Sunghoon Choi, Hyemi Kim, Zhen Chao, Seungyeon Choi, Hee‐Joung Kim

**Affiliations:** ^1^ Department of Radiation Convergence Engineering College of Health Science Yonsei University Wonju Gangwon Korea; ^2^ Department of Radiological Science College of Health Science Yonsei University Wonju Gangwon Korea

**Keywords:** dose reduction, ROI reconstruction, Tomosynthesis

## Abstract

Dose reduction techniques have been studied in medical imaging. We propose shutter scan acquisition for region of interest (ROI) imaging to reduce the patient exposure dose received from a digital tomosynthesis system. A prototype chest digital tomosynthesis (CDT) system (LISTEM, Wonju, Korea) and the LUNGMAN phantom (Kyoto Kagaku, Japan) with lung nodules 8, 10, and 12 mm in size were used for this study. A total of 41 projections with shutter scan acquisition consisted of 21 truncated projections and 20 non‐truncated projections. For comparison, 41 projections using conventional full view scan acquisition were also acquired. Truncated projections obtained by shutter scan acquisition were corrected by proposed image processing procedure to remove the truncation artifacts. The image quality was evaluated using the contrast to noise ratio (CNR), coefficient of variation (COV), and figure of merit (FOM). We measured the dose area product (DAP) value to verify the dose reduction using shutter scan acquisition. The ROI of the reconstructed image from shutter scan acquisition showed enhanced contrast. The results showed that CNR values of 8 and 12 mm lung nodules increased by 6.38% and 21.21%, respectively, and the CNR value of 10 mm lung nodule decreased by 3.63%. COV values of the lung nodules were lower in a shutter scan image than in a full view scan image. FOM values of 8, 10, and 12 mm lung nodules increased by 3.06, 2.25, and 2.33 times, respectively. This study compared the proposed shutter scan and conventional full view scan acquisition. In conclusion, using a shutter scan acquisition method resulted in enhanced contrast images within the ROI and higher FOM values. The patient exposure dose of the proposed shutter scan acquisition method can be reduced by limiting the field of view (FOV) to focus on the ROI.

## 1 INTRODUCTION

Dose reduction techniques have been of great interest in medical applications, and the digital tomosynthesis system has been studied to reduce the exposure dose. Gennaro et al. (2010) reported a comparison of the clinical performance of digital breast tomosynthesis (DBT) and digital mammography. This paper shows how DBT can be applied to actual clinical practice and has an expected effect in medical diagnostic image.^1^ Choi et al. (2017) reported the development of a prototype chest digital tomosynthesis (CDT) R/F system for the diagnosis of pulmonary nodules.^2^ These results demonstrate the clinical applicability of CDT and introduce low dose reconstitution method for clinical application. These studies emphasize the need for research how to reduce the dose of the patient for clinical application of digital tomosynthesis system. Diagnostically important information is often concentrated in a region of interest (ROI) within a reconstructed three‐dimensional (3D) image. Hence, ROI reconstruction techniques are considered reasonable dose reduction methods.

ROI reconstruction techniques have been studied mainly for computed tomography (CT) applications. Among them, Zhang et al. (2009) reported the study of ROI reconstruction using a two‐step filtering‐based iterative image reconstruction algorithm.^3^ This paper suggests that ROI reconstruction can be a useful method for clinical diagnosis. If the ROI reconstruction method is applied to a digital tomosynthesis system, a larger dose reduction effect can be expected. Recently, studies of ROI reconstruction have been studied for digital tomosynthesis system. Park et al. (2017) reported the ROI reconstruction method for digital tomosynthesis system by using scout view information to improve ROI reconstructed images.^4^ According to following these studies, limiting the field of view (FOV) can improve internal image quality of ROI and reduce the patient exposure, so the ROI reconstruction can have the possibility of clinical application.

Projections obtained using ROI imaging are truncated images. A reconstructed image with truncated projections has truncation artifacts, which degrade the quality of the reconstructed image. Thus, the accuracy of the ROI reconstruction method by the conventional algorithm may be insufficient, especially for clinical diagnosis.^5^, ^6^, ^7^, ^8^, ^9^, ^10^, ^11^ Several studies have examined artifact reduction in images reconstructed from truncated projections.^12^, ^13^, ^14^, ^15^, ^16^, ^17^, ^18^, ^19^, ^20^ Ruchala et al. (2002) reported an algorithm to reduce truncation artifacts caused by the limited field of view using *a priori* information.^21^ Dennerlein et al. (2013) suggested the approximate truncation robust computed tomography (ATRACT) filter method to reduce truncation artifacts in reconstructed images and improve the accuracy of the image.^22^

However, most studies do not focus on improvements in the image quality of the overall anatomy, particularly outside the ROI. We propose a shutter scan acquisition method based on ROI imaging using a digital tomosynthesis system. The projections obtained by the shutter scan acquisition method consist of both non‐truncated and truncated projections, so information outside the ROI can be obtained. The application of shutter scan acquisition for clinical practice is rarely studied. Therefore, this study is expected to be a feasibility study showing the clinical feasibility of ROI based imaging technology that can reconstruct the outside the ROI information applied to digital tomosynthesis system.

In this study, the shutter scan acquisition method for advanced ROI imaging was proposed to reduce the patient exposure dose received from a digital tomosynthesis system. The reconstructed image from shutter scan acquisition was corrected using the proposed image processing to remove the truncation artifacts. We applied shutter scan acquisition to a digital tomosynthesis system and evaluated the quality of the reconstructed image. The purpose of this study was to investigate the feasibility of proposed shutter scan acquisition for a digital tomosynthesis system.

## 2 MATERIALS AND METHODS

### 2.A Experimental set‐up

**Figure 1:**
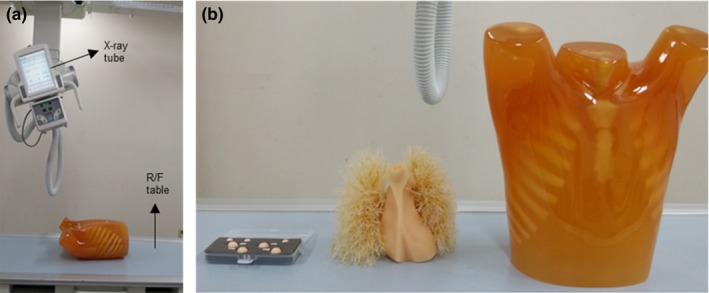
(a) Prototype chest digital tomosynthesis system, (b) lung nodule and LUNGMAN phantom.

We used a prototype chest digital tomosynthesis (CDT) R/F system (LISTEM, Wonju, Korea).The source‐to‐detector distance (SDD) and the source‐to‐object distance (SOD) were 1100 mm and 1000 mm, respectively. The X ray tube moved linearly at a speed of 160 mm/s. The phantom used in this study was a multipurpose chest phantom (LUNGMAN, Kagaku, Japan) with a normal anatomical structure of the human chest and lung nodules of 8, 10, and 12 mm. A photograph of the prototype CDT system and a LUNGMAN phantom with a lung nodule are shown in Fig. 1. Table 1 lists the major acquisition parameters of the prototype CDT system.

**Table 1:** Specifications of the prototype chest digital tomosynthesis system

Source to detector distance	1000–1500 mm
Isocenter to detector distance	100 mm
Detector information	
Pixel array	1440 × 1440 (43 × 43 cm^2^)
Pixel pitch	0.296 mm
Dynamic range	14 bit with ADC (Analog‐to digital converters)
X ray tube	40–120 kVp (voltage range) 10–500 mA (current range) 10 ms (exposure duration)
Number of projections (angular range)	41 projections (40°)
Reconstruction method	Reconstruction method –’FBP (Filtered back projection)’ Filter –’Ramp‐lak’

**Figure 2:**
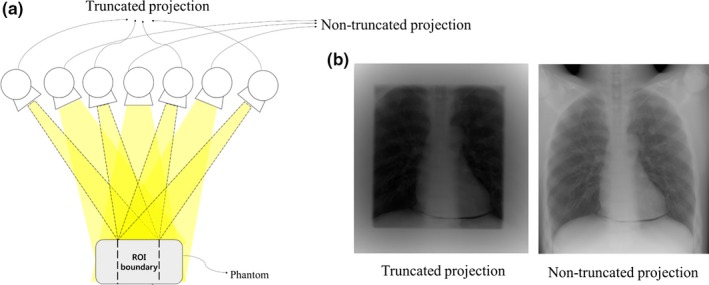
Illustration of shutter scan acquisition in a digital tomosynthesis system: (a) schematic diagram of shutter scan acquisition, (b) projections obtained using shutter scan acquisition.

A total of 41 projections were obtained using the shutter scan acquisition method; they consisted of 21 truncated projections and 20 non‐truncated projections over a 40° angular range. The illustration of the shutter scan acquisition is presented in Fig. 2. For comparison, we also obtained projections of the conventional full view scan, which consisted of 41 non‐truncated projections.

### 2.B Image processing

**Figure 3:**
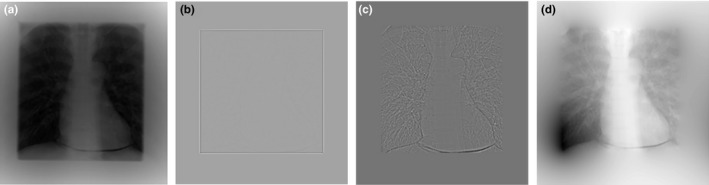
Projection images with step‐by‐step image processing: (a) truncated projection image, (b) after the Laplace operator, (c) after removing the edge, (d) after inverse Laplace operator.

**Figure 4:**
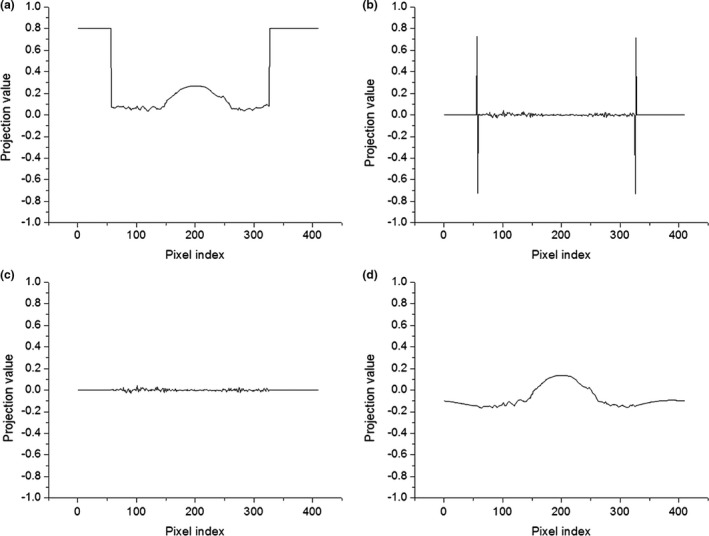
Line profiles of projection images during image processing: (a) truncated projection image, (b) after application of the Laplace operator, (c) after removing the edge, (d) after inverse Laplace operator.

The proposed shutter scan acquisition method contained truncated projections. The truncated portion of the projections caused a truncation artifact in the reconstructed image. An image processing procedure is required for the shutter scan acquisition method. We corrected the truncated projections using proposed image processing procedure as illustrated in Figs. 3 and 4. Two‐dimensional (2D) Laplace operator in eq. (1) detected the edge caused by the truncation. To remove the edge, the pixel values of the detected edges were linearly interpolated using the pixel values around the edge. After removing the edges, we performed inverse Laplace transform on the image.

**Figure 5:**
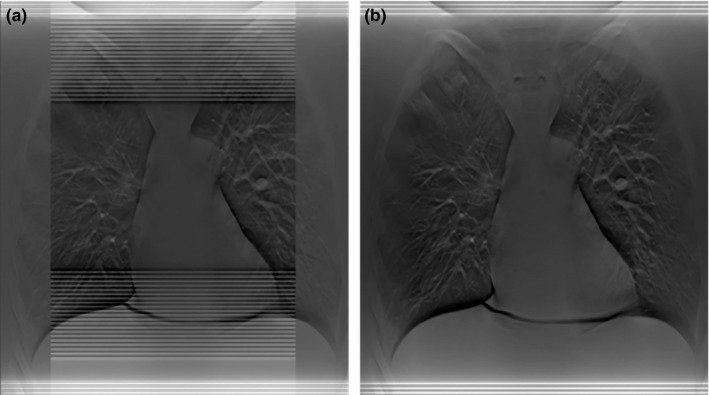
Reconstructed images using shutter scan acquisition in a digital tomosynthesis system: (a) before image processing, (b) after image processing.

The 2D Laplace operator can be expressed as follows (1):

(1) $$g_{2}\left(\lambda ,u,v\right)=\left(\frac{\partial^{2}}{\partial u^{2}}+\frac{\partial^{2}}{\partial v^{2}}\right)g_{1}\left(\lambda ,u,v\right)$$

where $g_{1}\left(\lambda ,u,v\right)$ is the projection at location (λ,u,v) and $g_{2}\left(\lambda ,u,v\right)$ is the projection after applying the Laplace operator. Three‐point second derivatives along *u* and *v* were used for discretization of the Laplace operation. The image‐processed projections were used to obtain the reconstructed images by applying the FBP algorithm. After removing the truncated portion, we reduced the truncation artifact in the reconstructed images as shown in Fig. 5.

### 2.C Image reconstruction process

A small number of projections are used to obtain reconstructed image from a limited angle of a digital tomosynthesis system. Existing reconstruction algorithms in digital tomosynthesis system can be divided into three categories: back projection (BP), filtered back projection (FBP), and iterative reconstruction algorithm.^23^ In this study, we used FBP algorithm because it is a commonly used reconstruction algorithm in clinical practice and takes least computation time.

**Figure 6:**
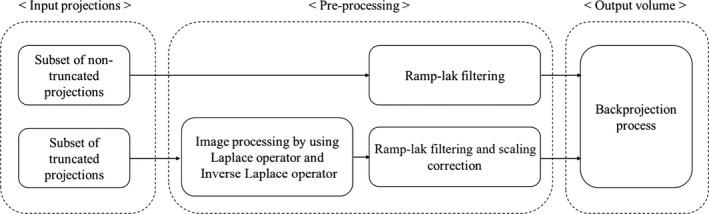
Flowchart of image reconstruction algorithm process in shutter scan acquisition.

Projections obtained through shutter scan acquisition consist of non‐truncated projections and truncated projections. After applying pre‐processing on subset of non‐truncated projections and subset of truncated projections respectively, we performed FBP reconstruction. A flowchart for the overall reconstruction process is shown in Fig. 6. Subset of non‐truncated projections was simply back projected after applying a ramp‐lak filter. Subset of truncated projections was processed by proposed image processing using Laplace operator and inverse Laplace transform, then ramp‐lak filter was applied. A detailed description of image processing is presented in session B above. Filtered truncated projection with the image processing yields a different scaling on projection intensity values compared with filtered non‐truncated projection. So we performed scaling correction based on pre‐computed linear regression model. Finally, back projection was performed after proposed scaling correction.

Scaling correction was performed by the following eq. (2):

(2) $${PV^{\prime }}^{\left(truncated\right)}=\frac{PV^{\left(truncated\right)}-\mu^{\left(truncated\right)}}{\sigma^{\left(truncated\right)}}\sigma^{\left(non-truncated\right)}+\mu^{\left(non-truncated\right)}$$

where ${PV^{\prime }}^{\left(truncated\right)}$ and $PV^{\left(truncated\right)}$ denote the corrected truncated projection and uncorrected truncated projection, $\mu^{\left(truncated\right)}$ and $\sigma^{\left(truncated\right)}$ denote the mean and the standard deviation of a region of interest in truncated projection. $\mu^{\left(non-truncated\right)}$ and $\sigma^{\left(non-truncated\right)}$ denote the mean and the standard deviation of a region of interest in non‐truncated projection which is nearby $PV^{\left(truncated\right)}$.

### 2.D Data analysis

The reconstructed image had a volume of 1000 × 1000 × 50 voxels. We set the ROIs and background on the 37th slice. In this study, image quality was investigated using the contrast‐to‐noise ratio (CNR), coefficient of variation (COV), and figure of merit (FOM) value. First, the CNR values of the lung nodules were calculated to compare the shutter scan with the full view scan. Three lung nodule regions were included in the ROI, and the nearest section was placed in the background to obtain the CNR values. This factor is a useful physical parameter to evaluate the image quality and was calculated by eq. (3):

(3) $$\text{CNR}=\frac{{\text{ROI}}_{\mathrm{organ}}-{\text{ROI}}_{\mathrm{background}}}{\sqrt{\frac{1}{2}\times \left({\text{SD}}_{\mathrm{organ}}^{2}+{\text{SD}}_{\mathrm{background}}^{2}\right)}}$$

where ROI_organ_ and ROI_background_ are the mean values, and SD_organ_ andSD_background_ are the standard deviations of the object and background regions, respectively.

Second, the COV, which represents the image noise property, was defined as the ratio of the standard deviation to the mean as described by eq. (4):

(4) $$\text{COV}=\frac{{\text{SD}}_{\mathrm{organ}}}{{\text{ROI}}_{\mathrm{organ}}}\times 100$$

To compare the differences between the two acquisition methods, both the CNR and COV values were normalized.

**Figure 7:**
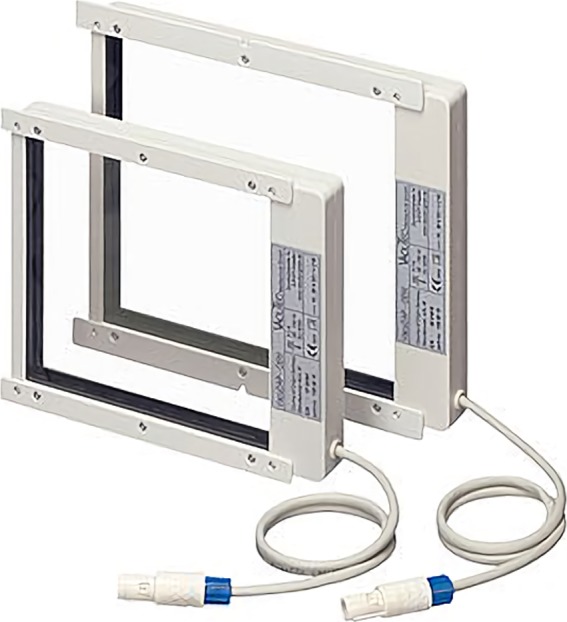
VacuDAP meter (VacuTec Meßtechnik GmbH, Dresden, Germany).

Third, the total dose area product (DAP) values were measured and compared. This was to assess how much the patient's area dose could be reduced using shutter scan acquisition. We measured the DAP values using a DAP meter (Fig. 7) (VacuTec Meßtechnik GmbH, Dresden, Germany).

In addition, the FOM values of the three lung nodules were calculated to quantitatively evaluate the image quality relative to the dose. The total effective dose was used as the dose value to calculate the FOM. We estimated the effective patient dose in the digital tomosynthesis system using PCXMC 2.0 software (STUK, Helsinki, Finland), which can simulate the effective dose based on a Monte Carlo simulation with publication 103 of the ICRP.^24^, ^25^, ^26^

The FOM values were calculated by the following eq. (5):

(5) $$\text{FOM}=\frac{{\text{CNR}}^{2}}{\text{effective dose}}$$

## 3 RESULTS

**Figure 8:**
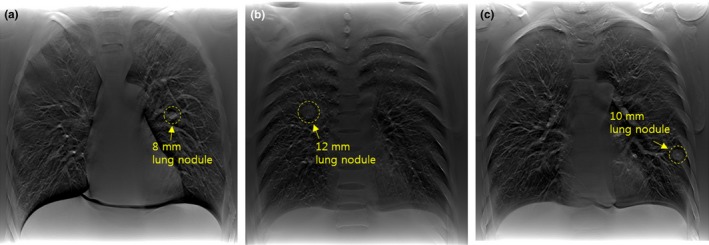
Reconstructed images of the region containing lung nodules in the LUNGMAN phantom: (a) reconstructed image of the 37th slice (within the ROI), (b) reconstructed image of the 25th slice (within the ROI), (c) reconstructed image of the 28th slice (outside the ROI).

**Figure 9:**
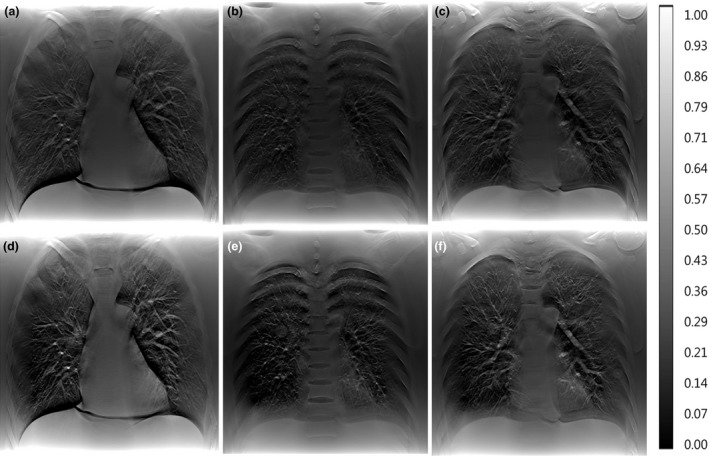
Reconstructed images of the LUNGMAN phantom: (a) full view of the 37th slice, (b) full view of the 25th slice, (c) full view of the 28th slice, (d) shutter scan of the 37th slice, (e) shutter scan of the 25th slice, (f) shutter scan of the 28th slice.

**Figure 10:**
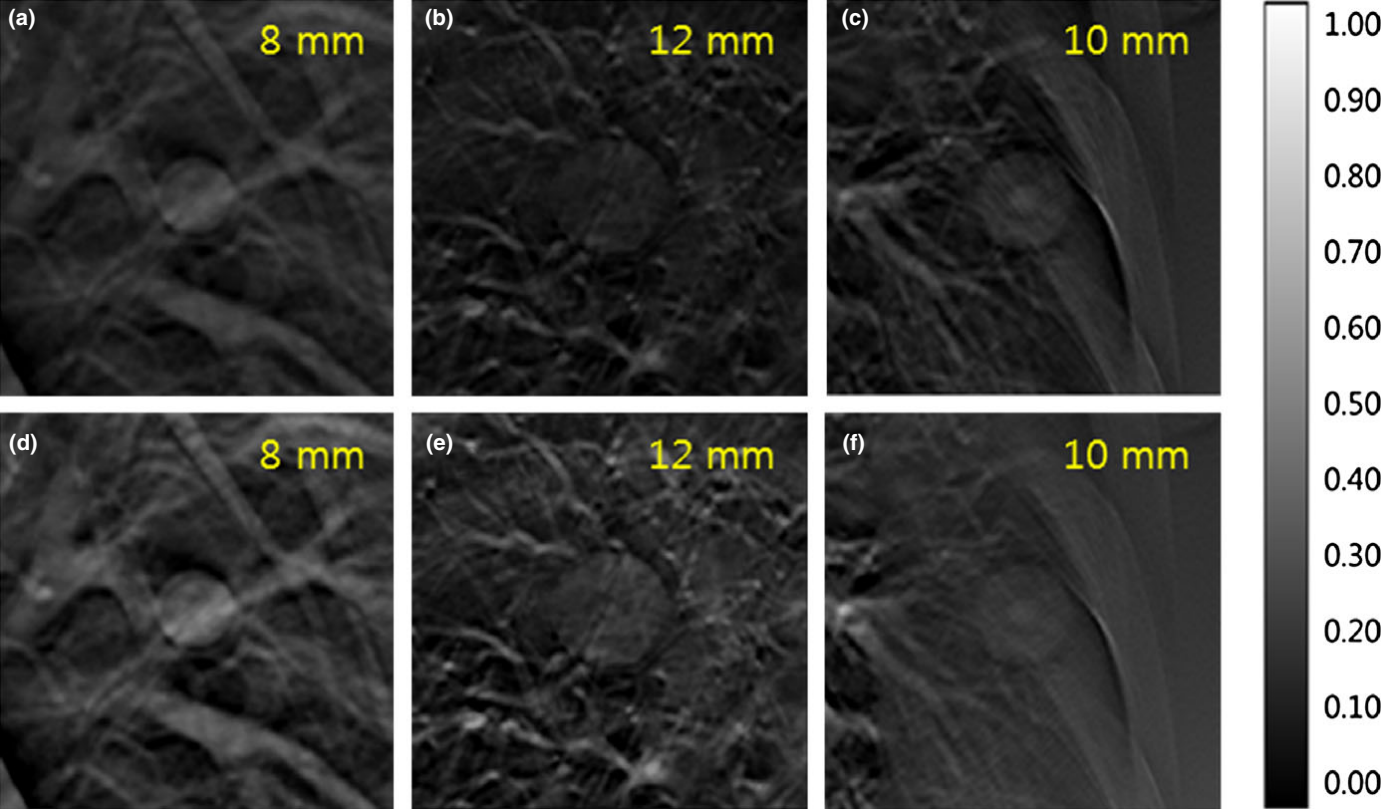
Three reconstructed, enlarged images of lung nodules: (a) full view of the 37th slice, (b) full view of the 25th slice, (c) full view of the 28th slice, (d) shutter scan of the 37th slice, (e) shutter scan of the 25th slice, (f) shutter scan of the 28th slice.

Some projections obtained by shutter scan acquisition were truncated images, and the reconstructed image without correction contained truncation artifacts. After applying proposed image processing, the reconstructed image did not contain truncation artifacts. The corrected reconstructed image showed better image quality compared with the reconstructed image using a conventional full view scan. Figure 8 shows the location of each lung nodule in the reconstructed image. The reconstructed images of the LUNGMAN phantom are shown in Fig. 9. A reconstructed, enlarged image of each lung nodule is presented in Fig. 10. The ROI of the reconstructed image showed enhanced contrast. However, the lung nodule outside the ROI did not.

**Figure 11:**
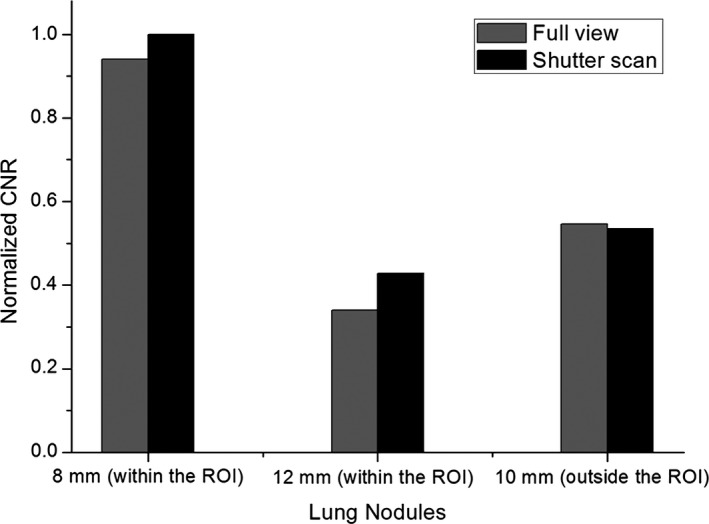
Normalized CNR values in reconstructed images with 41 projections using full view or shutter scan acquisition.

**Figure 12:**
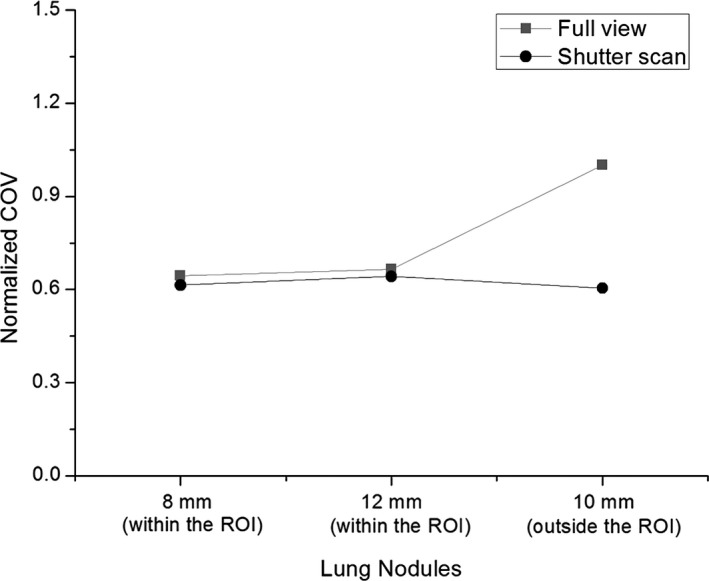
Normalized COV values in the reconstructed images with 41 projections using full view or shutter scan acquisition.

For quantitative analysis, CNR and COV values of three lung nodules were calculated and compared. The 8 and 12 mm lung nodules within the ROI showed higher CNR values with shutter scan acquisition than with full view acquisition. The 10 mm lung nodule outside the ROI showed a slightly lower CNR value with shutter scan acquisition than with full view acquisition, as presented in Fig. 11. CNR values of 8 and 12 mm lung nodules increased by 6.38% and 21.21%, respectively, and the CNR of 10 mm lung nodule decreased by 3.63%. Figure 12 shows that all COV values of all lung nodules with shutter scan are lower than with full view scan.

**Figure 13:**
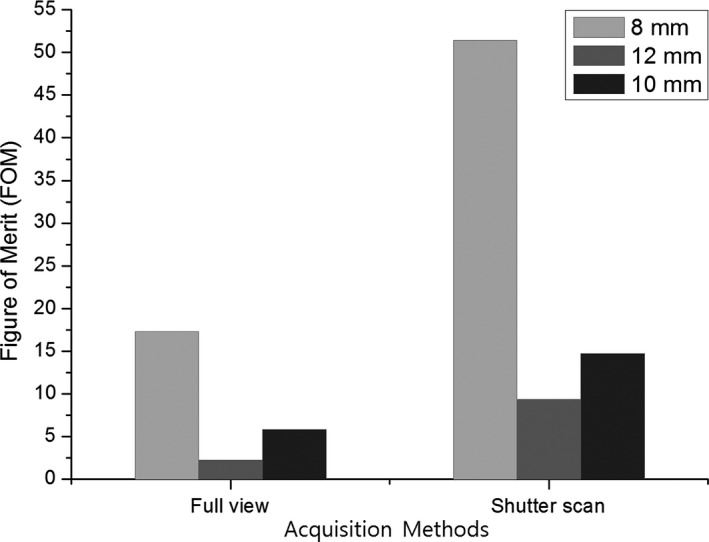
Figure of merit (FOM) values of three lung nodules in reconstructed images using full view or shutter scan acquisition.

Furthermore, DAP values were compared to verify the dose reduction. We also calculated the FOM values to see the performance of the image quality relative to its exposure dose. The results of the DAP evaluation (Table 2) showed that the area dose decreased by 36.36% using shutter scan acquisition compared to full view acquisition. All lung nodules showed higher FOM values with shutter scan acquisition than with full view acquisition, as presented in Fig. 13. FOM values of 8, 10 and 12 mm lung nodules increased by 3.06, 2.25, and 2.33 times, respectively.

**Table 2:** Total of DAP values in two different acquisition methods

	Full view acquisition	Shutter scan acquisition
DAP value (μGy·m^2^)	209.8	133.5

## 4 DISCUSSION

Various methods for reduction in the patient exposure dose during imaging have been widely studied. ROI reconstruction techniques have been the primary focus, and there have been many attempts to improve the image quality.^13^, ^21^ However, existing studies on the reconstruction of the image outside of the ROI are limited. The area outside the ROI is important; it reflects anatomical information for accurate clinical diagnosis. The proposed shutter scan acquisition method can reconstruct the area outside the ROI. It can also increase the image quality inside the ROI and reduce the patient exposure dose in comparison to the conventional full view scan.

Our results suggest that shutter scan acquisition results in better image quality than the conventional full view scan. In addition, these results indicate that shutter scan acquisition can be used to reconstruct the image both outside and inside the ROI. We quantitatively evaluated both the proposed shutter scan acquisition and the conventional full view scan acquisition, and verified the value of the shutter scan acquisition. The CNR evaluation demonstrates that the contrast in the ROI improved. Since smoothing effect of proposed image processing using Laplace and inverse Laplace operator has reduced image noise, CNR values were improved. Another expected reason of increased CNR values is because the amount of scatter radiation reaching the detector decreases as the field of view decreases. But, further study is needed to verify how quantitatively the amount of scatter decreases as the field of view decreases.

Although CNR values in the ROI have increased, there was little effect outside the ROI, so it is necessary to check the position of the lesion to determine the ROI for shutter scan acquisition. Outside the ROI, CNR improvement is difficult due to the lack of image information acquired by shutter scan acquisition compared with the full view scan. Likewise, the COV evaluation demonstrates the significant advantage of shutter scan acquisition in noise level reduction. The decreased COV value is a reason for the improved CNR in the ROI. In particular, the noise level of the lung nodule was greatly reduced outside the ROI. The results also demonstrate that shutter scan acquisition can have a significant benefit in terms of patient exposure dose. The results of the FOM demonstrate that shutter scan acquisition achieves enhanced contrast image within the ROI while reducing the exposure dose received by the patient.

## 5 CONCLUSIONS

This study compared the proposed shutter scan and conventional full view scan. In conclusion, reconstructed images using shutter scan acquisition using a digital tomosynthesis system resulted in better image quality with higher FOM values. In addition, it is expected that the patient exposure dose can be reduced by limiting the field of view (FOV) to focus on the ROI in the proposed shutter scan acquisition method. To develop the proposed shutter scan acquisition, it is necessary to verify the clinical value through a quantitative dose evaluation study. An optimization study is nawrassseeded to investigate the optimal acquisition parameters for shutter scan acquisition. Furthermore, a study of scatter radiation analysis is also needed to clarify the cause of improved image quality using shutter scan acquisition. We are going to also conduct further study of the clinical application for proposed shutter scan acquisition method to demonstrate applicability of this method for clinical cases.

## CONFLICT OF INTEREST

The authors declare no conflict of interest.

## References

[1] Gennaro G . Digital breast tomosynthesis versus digital mammography: a clinical performance study. Eur Radiol. 2010;20:1545–1553. 20033175 10.1007/s00330-009-1699-5

[2] Choi S . Development of a prototype chest digital tomosynthesis (CDT) R/F system with fast image reconstruction using graphics processing unit (GPU) programming. Nucl Instrum Methods Phys Res A. 2017;848:174–181.

[3] Zhang H , Li L , Yan B , Wang L , Cai A , Hu G . A two‐step filtering‐based iterative image reconstruction method for interior tomography. J Xray Sci Technol. 2016;24:733–747. 27392828 10.3233/XST-160584

[4] Park S . Scout‐view assisted interior digital tomosynthesis (iDTS) based on compressed‐sensing theory. Radiat Phys Chem. 2017;141:29–35.

[5] Bracewell RN , Wernecke SJ . Image reconstruction over a finite field of view. J Opt Soc Am. 1975;65:1342–1346.

[6] Gore JC , Leeman S . The reconstruction of objects from incomplete projections. Phys Med Biol. 1980;25:129. 7360784 10.1088/0031-9155/25/1/012

[7] Tofts PS , Gore JC . Some sources of artifact in computed‐tomography. Phys Med Biol. 1980;25:117. 7360783 10.1088/0031-9155/25/1/011

[8] Sankar PV , Nalcioglu O , Sklansky J . Undersampling errors in region‐of‐interest tomography. IEEE Trans Med Imag. 1982;1:168–173. 10.1109/TMI.1982.4307568 18238271

[9] Louis A , Rieder A . Incomplete data problems in X‐ray computerized tomography. II. Truncated projections and region‐of‐interest tomography. Numer Math. 1989;56:371–383.

[10] Lewitt RM . Processing of incomplete measurement data in computed tomography. Med Phys. 1979;6:412–417. 492075 10.1118/1.594519

[11] Cho S , Pearson E , Pelizzari CA , Pan X . Region‐of‐interest image reconstruction with intensity weighting in circular cone‐beam CT for image‐guided radiation therapy. Med Phys. 2009;36:1184–1192. 19472624 10.1118/1.3085825 PMC2673679

[12] Wagner W . Reconstructions from restricted region scan data – new means to reduce the patient dose. IEEE Trans Nucl Sci. 1979;26:2866–2869.

[13] Nalcioglu O , Cho ZH , Lou RY . Limited field of view reconstruction in computerized tomography. IEEE Trans Nucl Sci. 1979;26:546–551.

[14] Ogawa K , Nakajima M , Yuta S . A reconstruction algorithm from truncated projections. IEEE Trans Med Imag. 1984;3:34–40. 10.1109/TMI.1984.4307648 18234608

[15] Ohnesorge B , Flohr T , Schwarz K , Heiken JP , Bae KT . Efficient correction for CT image artifacts caused by objects extending outside the scan field of view. Med Phys. 2000;27:39–46. 10659736 10.1118/1.598855

[16] Hsieh J , Chao E , Thibault J , et al. A novel reconstruction algorithm to extend the CT scan field‐of‐view. Med Phys. 2004;31:2385–2391. 15487717 10.1118/1.1776673

[17] Chityala R , Hoffmann KR , Rudin S , Bednarek DR . Region of interest (ROI) computed tomography (CT): comparison with full field of view (FFOV) and truncated CT for a human head phantom. Proc Soc Photo Opt Instrum Eng. 2005;5745:583–590. 10.1117/12.595430 PMC3035320 21311728

[18] Hooper HR , Fallone BG . Technical note: sinogram merging to compensate for truncation of projection data in tomotherapy imaging. Med Phys. 2002;29:2548–2551. 12462721 10.1118/1.1514579

[19] Chen Z . Local volume reconstruction from width‐truncated cone‐beam projections by convolution backprojection. Opt Eng. 2008;47:017001.

[20] Wan X , Yi J , Zhang Z , Xiao W , Liu L . Lagrange interpolation reprojection‐revising reconstruction with incomplete data in optical computed tomography. Opt Eng. 2010;49:087001.

[21] Ruchala KJ , Olivera GH , Kapatoes JM , Reckwerdt PJ , Mackie TR . Methods for improving limited field‐of‐view radiotherapy reconstructions using imperfect *a priori* images. Med Phys. 2002;29:2590–2605. 12462726 10.1118/1.1513163

[22] Dennerlein F , Maier A . Approximate truncation robust computed tomography. Phys Med Biol. 2013;58:6133–6148. 23941816 10.1088/0031-9155/58/17/6133

[23] Tao Wu . A comparison of reconstruction algorithms for breast tomosynthesis. Med Phys. 2004;31:2636–2647. 15487747 10.1118/1.1786692

[24] Tapiovaara M , Lakkisto M , Servomaa A . PCXMC: a PC‐based Monte Carlo program for calculating patient doses in medical X‐ray examinations. *STUK‐A1391997*, Finland; 1997.

[25] Sabol JM . A Monte Carlo estimation of effective dose in chest tomosynthesis. Med Phys. 2009;36:5480–5487. 20095260 10.1118/1.3250907

[26] ICRP . The 2007 Recommendations of the International Commission on Radiological Protection. ICRP Publication 103. Ann. ICRP 37 (2‐4); 2007. 10.1016/j.icrp.2007.10.003 18082557

